# A combined biomarker approach for characterising extracellular matrix profiles in acute myocardial infarction

**DOI:** 10.1038/s41598-021-92108-z

**Published:** 2021-06-16

**Authors:** Morgane M. Brunton-O’Sullivan, Ana S. Holley, Kathryn E. Hally, Gisela A. Kristono, Scott A. Harding, Peter D. Larsen

**Affiliations:** 1 Department of Surgery and Anaesthesia, University of Otago, Wellington, 6242 New Zealand; 2 Wellington Cardiovascular Research Group, University of Otago, Wellington, 6242 New Zealand; 3 School of Biological Sciences, Victoria University of Wellington, Wellington, 6012 New Zealand; 4 Department of Cardiology, Wellington Regional Hospital, Wellington, 6242 New Zealand

**Keywords:** Myocardial infarction, Prognostic markers

## Abstract

Extracellular matrix (ECM) biomarkers are useful for measuring underlying molecular activity associated with cardiac repair following acute myocardial infarction (AMI). The aim of this study was to conduct exploratory factor analysis (EFA) to examine the interrelationships between ECM biomarkers, and cluster analysis to identify if distinct ECM profiles could distinguish patient risk in AMI. Ten ECM biomarkers were measured from plasma in 140 AMI patients: MMP-2, -3, -8, -9, periostin, procollagen I N-Terminal propeptide, osteopontin, TGF-β1, TIMP-1 and -4. EFA grouped eight ECM biomarkers into a two-factor solution, which comprised three biomarkers in Factor 1 and five biomarkers in Factor 2. Notably, ECM biomarkers were not separated based on biological function. Cluster analysis grouped AMI patients into three distinct clusters. Cluster One (n = 54) had increased levels of MMP-8, MMP-9, and TGF-B1. Cluster Two (n = 43) had elevated levels of MMP-2, MMP-3, osteopontin, periostin and TIMP-1, and increased high-sensitivity troponin T and GRACE scores. Cluster Three (n = 43) had decreased levels of ECM biomarkers. Circulating ECM biomarkers demonstrated collinearity and entwined biological functions based on EFA analysis. Using cluster analysis, patients with similar clinical presentations could be separated into distinct ECM profiles that were associated with differential patient risk. Clinical significance remains to be determined.

## Introduction

The cardiac extracellular matrix (ECM) provides structural organisation to the myocardium, and facilitates electrical transduction, molecular signalling and intercellular communication^1,2^. Following ischaemic injury, as observed during myocardial infarction (MI), the ECM is altered to compensate for the negligible regeneration capacity of the adult mammalian heart^3^. This process is termed ECM remodeling^4^, and describes the replacement of damaged tissue with non-contractile scar^1^.

The ECM response post-MI is a coordinated process orchestrated by multiple cellular and molecular factors^2,5^. For example, matrix metalloproteinases (MMPs) are upregulated during the early inflammatory phase to mediate the removal of cellular debris at the infarct site^6^. This is followed by deposition of ECM during the proliferative phase which is regulated by signalling molecules, such as matricellular proteins and growth factors^3^. Indeed, murine models of MI have demonstrated the importance of a timely and regulated ECM response for optimal global myocardial repair^7–10^.

Measuring the ECM response in humans is considerably more challenging, as access to the myocardium is both invasive and clinically non-viable. However, measuring circulating ECM biomarkers provides an opportunity to capture ECM activity^5^. Previous research has investigated single ECM biomarkers and linked circulating levels to myocardial remodeling outcomes post-MI^5,11–13^. While this remains important for translational research, it largely oversimplifies a complex pathophysiological process that is comprised of many individual components. A more comprehensive analysis of combined ECM biomarker activity has not yet been undertaken, and identifying techniques to capture the ECM response is required.

Two statistical approaches that can be used to understand such complexity are exploratory factor analysis (EFA) and cluster analysis. The primary purpose of EFA is to define the underlying structure of data based on correlations between variables^14,15^. In this context, it is a powerful tool to investigate interrelationships between circulating ECM biomarkers without prior assumptions of likely associations. Complimentary to this technique is cluster analysis, which can organise patients into distinct groups based on ECM biomarker levels. While EFA identifies groupings of variables, cluster analysis can separate patients with similar biomarker profiles by maximising homogeneity within a cluster and heterogeneity between clusters^16,17^.

Using these two techniques on a single AMI cohort provides an in-depth analysis of the ECM response in terms of both biomarker activity and patient profiles. This analysis will elucidate how we might capture the complexity of ECM biomarker interrelationships and identify whether there are patients with distinct ECM biomarker profiles. We believe that such an approach is required to investigate the state of ECM, prior to examining potential links to either short-term or long-term left ventricular (LV) remodeling.

In this study, we aimed to use combined statistical approaches to characterise ECM biomarkers in a cohort of AMI patients. Firstly, we employed EFA to investigate the underlying structure and interrelationships of 10 circulating ECM biomarkers. Secondly, we used hierarchical cluster analysis to identify whether distinct ECM profiles existed within the patient population based on the expression of these ECM biomarkers.

## Results

### Demographics and clinical characteristics

Baseline demographics and clinical characteristics of the 140 patients AMI patients are summarised in Table 1. The study population was 76.4% male with a mean age of 61 years and a mean BMI of 28.4. Of the patients in this cohort, 84.3% identified as European, 11.4% identified as Māori and Pasifika, and a further 4.3% identified as ‘Other’. Upon index admission, 44.3% of patients had hypertension, 54.3% had dyslipidaemia, 15.0% had diabetes and 22.9% were current smokers. Patients in this AMI cohort presented as Non-ST Elevation Myocardial Infarction (NSTEMI; 73.6%) and ST-Elevation Myocardial Infarction (STEMI; 26.4%).

**Table 1:** Baseline demographics of the study population.

Characteristics	Patients (n)
Age (mean, years)	61 ± 11
Male (n)	107 (76.4)
BMI (median, kg/m^2^)	28.4 (25.7–32.8)
**Ethnicity**	
European	118 (84.3)
Māori and Pasifika	16 (11.4)
Other	6 (4.3)
**Risk factors**	
Hypertension	62 (44.3)
Dyslipidaemia	76 (54.3)
Diabetes	21 (15.0)
Current Smoker	32 (22.9)
**Classification**	
NSTEMI	103 (73.6)
STEMI	37 (26.4)
**Biochemical measurements**	
MMP-2 (ng/mL)	106.92 (94.12–134.38)
MMP-3 (ng/mL)	10.15 (7.77–15.07)
MMP-8 (ng/mL)	0.41 (0.14–0.74)
MMP-9 (ng/mL)	17.31 (11.78–28.69)
Osteopontin (ng/mL)	36.71 (27.14–47.26)
Periostin (ng/mL)	85.25 (69.39–107.15)
PINP (ng/mL)	0.53 (0.30–0.97)
TGF-β1 (ng/mL)	4.41 (3.10–6.14)
TIMP-1 (ng/mL)	71.83 (54.37–85.37)
TIMP-4 (ng/mL)	2.70 (2.04–3.53)
High-sensitivity troponin T (ng/L)	382 (133–1,583)
GRACE score	122 (96–145)

Parametric continuous variables were expressed as mean ± SD and non-parametric continuous variables were expressed as median (IQR). Categorical variables were expressed as frequencies (percentages).

*BMI* body mass index, *NSTEMI* non-ST-elevation myocardial infarction, *STEMI* ST-elevation myocardial infarction, *GRACE* Global Registry of Acute Coronary Events.

### Correlations between extracellular matrix biomarkers

In total, 10 ECM biomarkers were measured in this study: matrix metalloproteinase (MMP) -2, -3, -8, -9, osteopontin, periostin, procollagen N-terminal propeptide (PINP), transforming growth factor beta 1 (TGF-β1), tissue inhibitor of matrix metalloproteinase (TIMP) -1 and -4. Blood samples were collected from patients on day three following hospital admission, and biomarkers were quantified in plasma using ELISA or Luminex assays.

**Figure 1:**
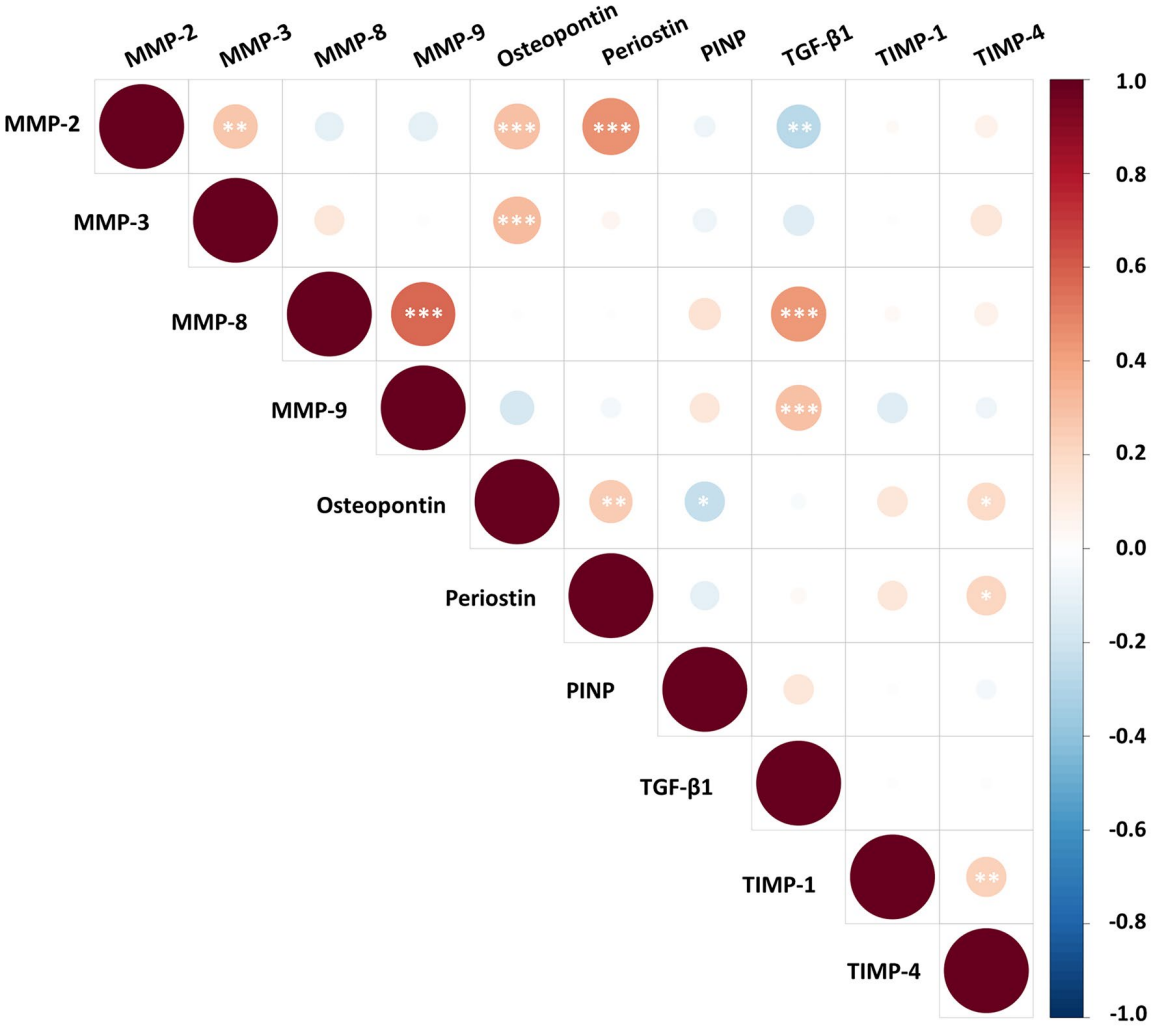
Correlation matrix between extracellular matrix biomarkers. Spearman’s Rank correlation was performed between extracellular matrix biomarker pairs. Red shading indicates positive correlations and blue shading indicates inverse correlations. Significance is shown by white asterisk symbols. *p < 0.05, **p < 0.001, ***p < 0.0001. This image was created using the corrplot package in R version 4.0.2, www.R-project.org.

The relationship between all ECM biomarkers was examined using Spearman’s Rank correlation. Of the 45 pairs examined, 13 significant correlations were observed (Fig. 1). These correlations were weak-to-moderate in strength, with the strongest correlation observed between MMP-8 and MMP-9 (r_s_ = 0.571, *p* < 0.0001). Of the 13 significant correlations, two inverse relationships were observed between osteopontin and PINP (r_s_ = − 0.225, *p* < 0.05) and TGF-β1 and MMP-2 (r_s_ = − 0.267, *p* = 0.001).

### Correlations between patient risk and extracellular matrix biomarkers

Peak high-sensitivity Troponin T (hs-TnT) can be used as a surrogate marker for infarct size^18^, while Global Registry of Acute Coronary Events (GRACE) scores estimates the probability of mortality within 6 months of hospital discharge^19^. The relationship between peak hs-TnT, GRACE scores and ECM biomarkers were examined using Spearman’s Rank correlation with weak-to-moderate correlations observed. For peak hs-TnT, three positive and significant correlations were observed and these were with MMP-8 (r_s_ = 0.172, *p* < 0.05), osteopontin (r_s_ = 0.341, *p* < 0.0001) and TIMP-1 (r_s_ = 0.206, *p* < 0.05) (see Supplementary Fig. S1). For GRACE scores, four positive significant correlations were observed and these were between MMP-2 (r_s_ = 0.250, *p* < 0.01), MMP-3 (r_s_ = 0.232, *p* < 0.01), osteopontin (r_s_ = 0.304, *p* < 0.001) and TIMP-4 (r_s_ = 0.277, *p* = 0.001) (see Supplementary Fig. S2). In addition, MMP-9 levels were inversely correlated with GRACE scores (r_s_ = − 0.208, *p* < 0.05) (see Supplementary Fig. S2).

### Relationship between clinical risk factors and extracellular matrix biomarkers

Significant relationships between ECM biomarkers and clinical characteristics are summarised in Table 2. Detailed correlation values and differences between clinical characteristics and ECM biomarkers are shown in Supplementary Tables S1 to S5 online.

**Table 2:** Summary of significant relationships between ECM biomarkers and clinical characteristics.

Clinical variable	MMP-2	MMP-3	MMP-8	MMP-9	Osteopontin	Periostin	PINP	TGF-β1	TIMP-1	TIMP-4
Age	0.295	0.216			0.298	0.231	− 0.197		0.194	0.429
Male		↑					↓		↓	↓
Hypertension						↑				↑
Dyslipidaemia							↑			
Diabetes										↑
STEMI					↑					

Only significant (*p* < 0.05) relationships are shown in this table. For values and levels of significance, refer to Supplementary Tables S1 to S5 online. Spearman’s Rank correlation was conducted between age and ECM biomarkers. The strength of significant correlations has been recorded. Mann–Whitney U testing was used to identify differences between categorical variables and ECM biomarkers. An arrow indicates a significant relationship, with the direction specifying if ECM biomarker levels were increased (↑) or decreased (↓) for the clinical variable measured.

When biomarkers were correlated with age, seven of the 10 pairs were statistically significant. These significant correlations were weak-to-moderate in strength, with the strongest correlation observed between age and TIMP-4 (r_s_ = 0.429, *p* < 0.01). A significant inverse relationship was also observed between age and PINP (r_s_ = − 0.197, *p* < 0.05). No significant correlations were observed between BMI and ECM biomarkers. Female patients had higher levels of PINP, TIMP-1 and TIMP-4 (all *p* < 0.05) when compared to male patients. In comparison, male patients had increased MMP-3 levels (*p* < 0.001) compared to females.

Patients presenting with NSTEMI had lower circulating levels of osteopontin (*p* < 0.05) compared to patients who presented with STEMI. Patients diagnosed with hypertension had increased levels of periostin (*p* < 0.05) and TIMP-4 (*p* < 0.01) when compared to non-hypertensive patients. Higher levels of TIMP-4 (*p* = 0.05) were observed in diabetic patients and higher levels of PINP (*p* < 0.05) were observed in patients diagnosed with dyslipidaemia. In this cohort, no differences were observed in ECM biomarker levels across ethnicities.

### Exploratory factor analysis

Exploratory Factor Analysis (EFA) was performed on 10 log-transformed ECM biomarkers using principle axis factoring with Oblimin rotation. Model fit was assessed using the Kaiser–Meyer–Olkin (KMO) measure which verified sampling adequacy with a value of 0.6 which is greater than the required threshold of 0.5. Bartlett’s Test for Sphericity, which assesses collinearity within a dataset, was significant for this study (*p* < 0.0001) and suggests EFA is an appropriate method to examine meaningful relationships between variables.

A factor represents a group of items that are highly interrelated. The strength in association between an item and the corresponding factor can be determined by the factor loading score. A factor loading closer to ± 1 demonstrates a strong relationship, and the squared factor loading is the amount of total variance that is accounted for by the factor. In this study, we have presented all variables which have factor loadings > 0.3, which accounts for approximately 10% of item variance.

EFA identified a two-factor solution which best described the relationship between ECM biomarkers in this AMI cohort. The rotated factor matrix, which describes the composition and loadings for each factor, is shown in Table 3. Factor 1 clustered three biomarkers with factor loadings greater than 0.3. MMP-8 and MMP-9 were highly correlated with Factor 1, while TGF-β1 only moderately contributed to Factor 1 which was demonstrated by a smaller factor loading. Factor 1 accounted for 15.88% of variance.

**Table 3:** The rotated factor matrix for circulating ECM biomarkers using EFA.

Variables	Factor 1 loadings	Factor 2 loadings
MMP-8	0.919	–
MMP-9	0.621	–
TGF-β1	0.448	–
MMP-2	–	0.606
Osteopontin	–	0.565
MMP3	–	0.499
Periostin	–	0.408
PINP	–	− 0.312
% of variance (rotated Sum of Squared loadings)	15.88	12.33

EFA performed on 10 log-transformed ECM biomarkers. Only factor loadings > 0.3 are presented.

In comparison, Factor 2 was comprised of five biomarkers with factor loadings greater than 0.3. MMP-2 and osteopontin significantly contributed to Factor 2. Comparably, MMP-3, periostin and PINP only had mid-range factor loadings between − 0.312 and 0.499. Factor 2 accounted for 12.33% of variance. In this EFA, PINP was the only biomarker with a negative factor loading and this suggests that lower levels of PINP result in positive factor loadings. No biomarkers were cross-correlated across factors and TIMP-1 and TIMP-4 did not contribute to either factor despite being included in the input for the EFA.

### Cluster analysis

**Figure 2:**
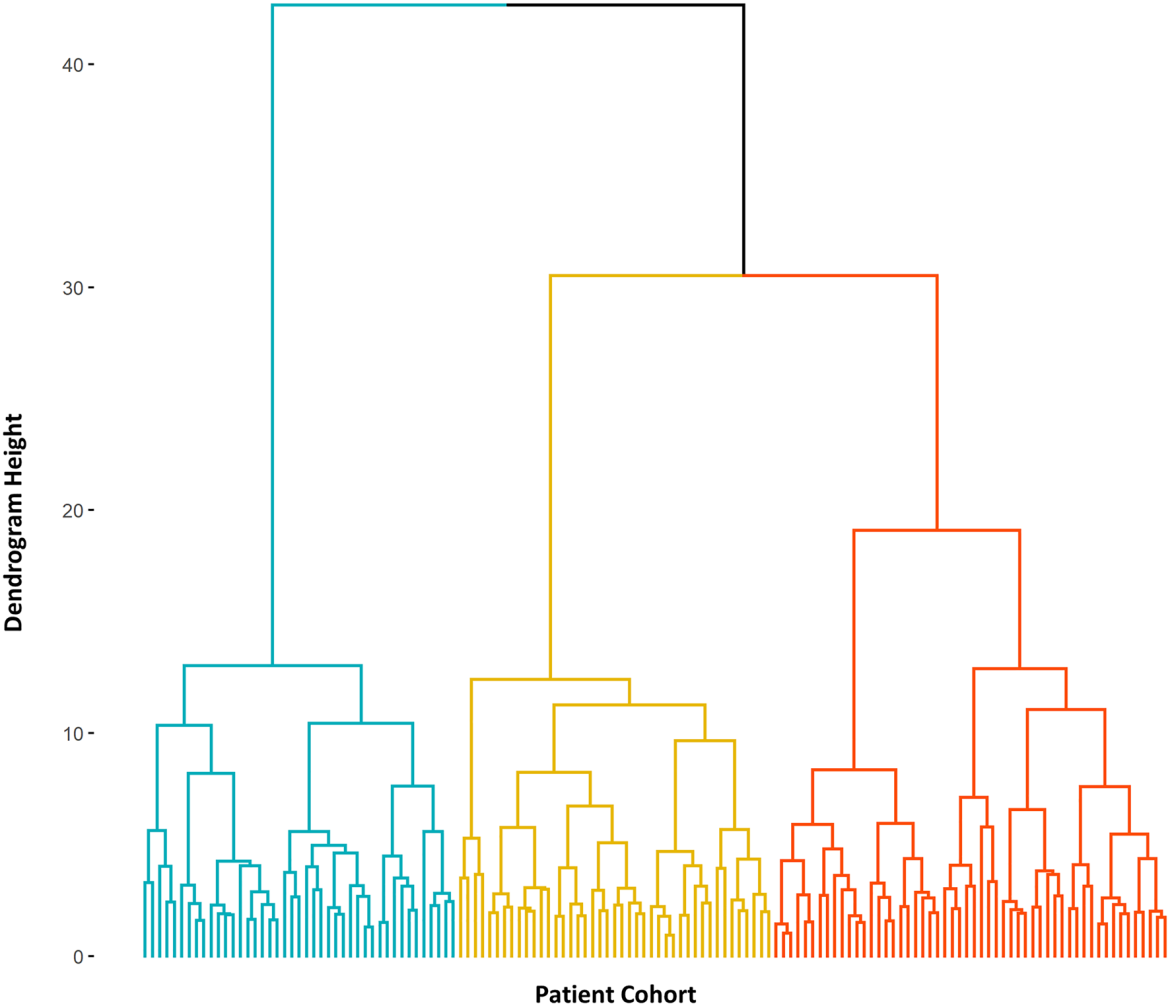
Dendrogram of hierarchical cluster analysis. Hierarchical cluster analysis identified three distinct groups of AMI patients based on ECM biomarker levels. This image was created using the factoextra package in R version 4.0.2, www.R-project.org.

**Figure 3:**
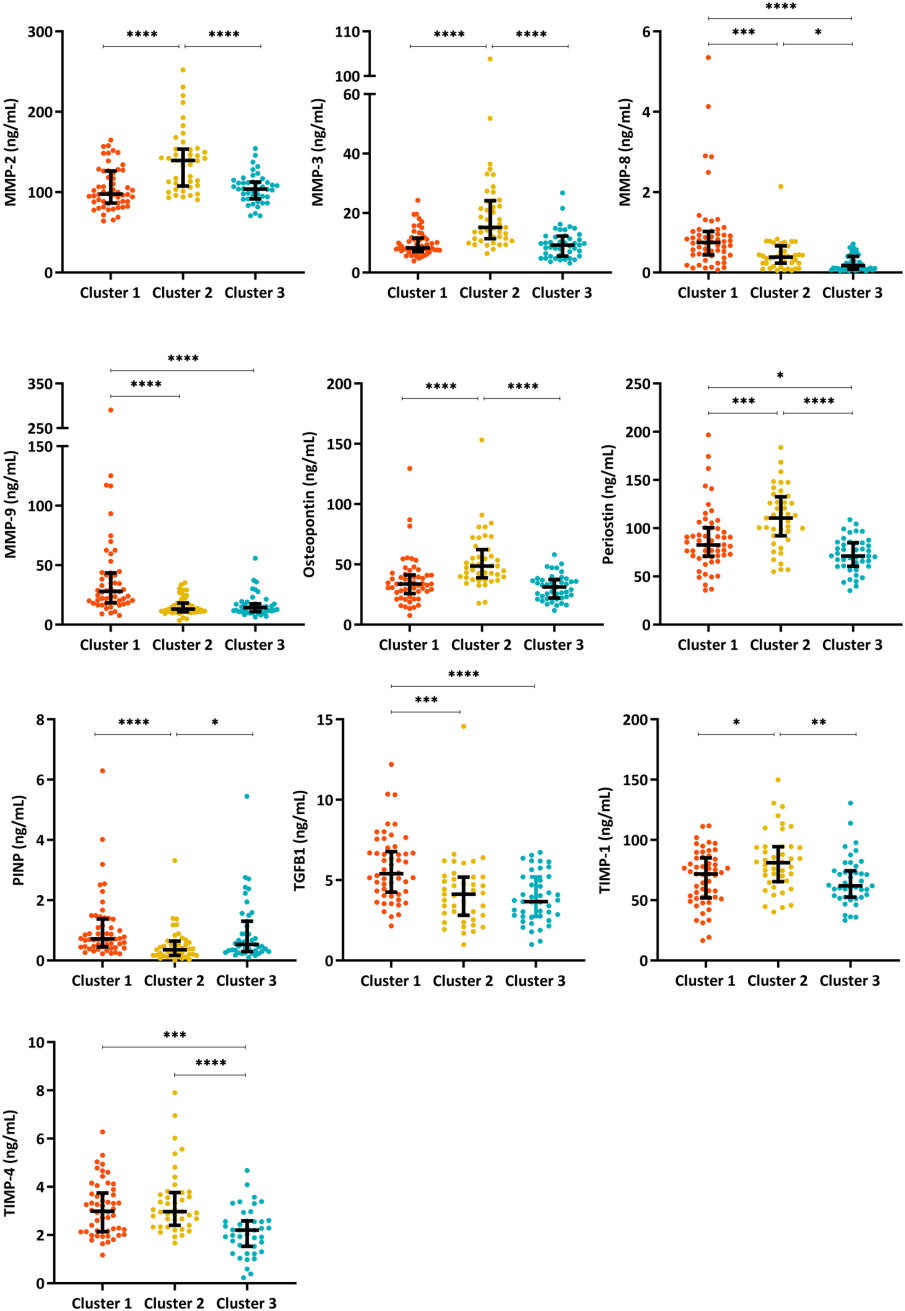
Extracellular matrix biomarker levels between clustered groups. Kruskal–Wallis test with Dunn’s multiple comparisons was performed to identify significant difference in ECM biomarker levels between patient clusters. Median (IQR) is plotted for each graph.*p < 0.05, **p < 0.01, ***p < 0.001, ****p < 0.0001. This image was created using GraphPad Prism software, version 7.04 for Windows, www.graphpad.com.

Using EFA analysis, we have shown that complex interrelationships exist between ECM biomarkers. To capture this complexity, we have employed cluster analysis to investigate ECM profiles in our AMI population. Hierarchical cluster analysis identified three patient groups with different ECM biomarker profiles. A dendrogram of patient clustering is shown in Fig. 2. Differences in clinical characteristics between clustered groups is shown in Table 4, and differences in ECM biomarker levels between clusters are shown in Fig. 3 and Supplementary Table S6 online.

**Table 4:** Patient demographics and clinical characteristics of clustered groups.

Characteristics	Cluster one (n = 54)	Cluster two (n = 43)	Cluster three (n = 43)
Age (median, years)	**60 (54**–**66)**	**67 (61**–75)**	**58 (47**–**66)**^**+++**^
Male (n)	39 (70.2)	36 (83.7)	32 (74.4)
BMI (median, kg/m^2^)	28.3 (25.9–32.7)	28.4 (25.1–32.9)	28.4 (25.1–32.5)
**Ethnicity**			
European	44 (81.5)	35 (81.4)	39 (90.7)
Māori & Pasifika	7 (13.0)	6 (13.9)	3 (6.9)
Other	3 (5.6)	2 (4.7)	1 (2.3)
**Risk factors**			
Hypertension	28 (51.9)	21 (48.8)	13 (30.2)
Dyslipidaemia	29 (53.7)	26 (60.5)	21 (48.8)
Diabetes	8 (14.8)	8 (18.6)	5 (11.6)
Current smoker	15 (27.8)	10 (23.3)	7 (16.3)
**Classification**			
NSTEMI	37 (68.5)	30 (69.8)	36 (83.7)
STEMI	17 (31.5)	13 (30.2)	7 (16.3)
**Patient risk**			
h s-Troponin T (ng/L)	458 (127.0–1527.5)	**644.0 (163**–**2700.0)**	**218.0 (105.5**–**683.5)**^**+**^
GRACE score	**117.1 ± 30.9**	136.3 ± 31.7**	**117.3 ± 33.2**^**+**^

Significant differences are shown in bold.

Continuous variables are reported as median (IQR). Kruskal–Wallis test with Dunn’s correction for multiple comparisons was used to compare continuous variables between clusters. *denotes significance compared to Cluster 1. ^+^denotes significance compared to Cluster 2. **p < 0.01, ^+^*p* < 0.05, ^+++^*p* < 0.001. Categorical variables are reported as frequency (percentage). Chi Square test was used to determine differences in categorical variables between clusters.

Cluster One (n = 54) comprised the largest patient group. Patients in Cluster One had significantly elevated levels of MMP-8, MMP-9 and TGF-β1 when compared to all other clusters. Levels of PINP and TIMP-4 were also increased when compared to patients in Cluster Two, while MMP-3 levels were significantly decreased.

Cluster Two (n = 43) comprised patients with an increased median age compared to Cluster One (*p* < 0.01) and Three (*p* < 0.001). Peak levels of MMP-2, MMP-3, osteopontin, periostin and TIMP-1 were observed in Cluster Two when compared to other clustered groups. Median levels of MMP-8 and TIMP-4 were increased when compared to Cluster Three, while PINP levels were significantly decreased. Patients in Cluster Two had significantly elevated GRACE scores when compared to patients in Cluster One (*p* < 0.01) and Cluster Three (*p* < 0.05). Peak hs-TnT levels were also significantly elevated in Cluster Two when compared to Cluster Three (*p* < 0.05).

Patients in Cluster Three (n = 43) demonstrated significantly lower median levels of most ECM biomarkers (Fig. 3 and Supplementary Table S6). Median levels of MMP-8, periostin and TIMP-4 were significantly reduced when compared to all clustered groups. Levels of MMP-2, MMP-3, osteopontin and TIMP-1 were significantly lower in patients from Cluster Three compared to Cluster Two, while PINP levels were increased. Median levels of MMP-9 and TGF-β1 were decreased in this group compared to Cluster One.

## Discussion

In this study, we have explored the complexity and heterogeneity of ECM biomarkers measured in AMI patients on day three following hospital admission. EFA demonstrated entwined and collinear interrelationships exist between ECM biomarkers. To account for this complexity, we applied cluster analysis to our AMI population and this identified three subgroups of patients that exhibited distinct ECM biomarker profiles with differential patient risk. These findings suggest that AMI patients can be partitioned into phenotypically distinct groups based on ECM biomarkers alone, and this provides an opportunity to discriminate between patients using a combined biomarker methodology.

To understand the complexity of interrelationships between measured ECM biomarkers in-depth, we employed EFA. This is an advanced statistical technique with the primary purpose of defining the underlying structure of data based on the correlations between variables^15^. This approach takes multidimensional data and reduces it to overarching latent variables, known as factors. Variables with high collinearity are grouped together in a single factor, and their factor loading value represents the strength of their relationship within a factor^14^.

In this study, a two-factor solution best described ECM biomarkers in our AMI cohort. Factor 1 was comprised of three ECM biomarkers. In this factor, MMP-8 had the highest loading value, and this was followed by MMP-9 and then TGF-β1. This composition could suggest that Factor 1 represents an ECM degradation phenotype, as MMPs are responsible for mediating the removal of cellular debris at the infarct site^20^, while TGF-β1 can activate these pathways as a multifunctional growth factor^21^. In comparison, Factor 2 comprised a larger number of ECM biomarkers with weak- to mid-range loadings. This suggests that a combination of ECM biomarkers jointly contributed to the composition of Factor 2, unlike Factor 1, which was mostly described by a single biomarker. Biomarkers with the strongest loadings in Factor 2 were MMP-2 and osteopontin, closely followed by MMP-3, periostin and PINP. Consequently, deciphering the latent variable represented by Factor 2 is more challenging due to the combination of ECM biomarkers included, which are not separated solely based on biological function.

This segues into the most important findings from EFA in this study. Firstly, EFA has confirmed that collinearity exists within the ECM biomarkers measured in this patient cohort. This is shown by the significant Bartlett’s Test for Sphericity which confirms interrelationships exist between measured variables and by the inclusion of multiple biomarkers within each factor. EFA has also importantly demonstrated that ECM biomarkers are not grouped solely on biological function. This is particularly evident in the distribution of MMPs, which are evenly spread across both factors. Overall, these findings suggest that ECM biomarker relationships are entwined and consequently measuring multiple ECM biomarkers may be important for capturing the complexity of these interrelationships.

While included in the analysis, both TIMP-1 and TIMP-4 were not represented by either factor upon EFA because their factor loadings were below 0.3. This suggests that TIMPs were not meaningfully captured by either factor^22^. All biomarker measurements recorded for this study were collected three days following hospital admission, and this time point may favour cardiac ECM degradation processes as tissue clearance dominates early repair^1^. Indeed, Factor 1 and Factor 2 both comprised biomarkers that represented ECM degradation activity. As TIMPs are associated with ECM deposition mechanisms due to their active role in MMP inhibition^23^, this may be a biological explanation for why these were not captured by the EFA factors. The time point of three days post-hospital admission was chosen as it is the latest measurement collected from hospital inpatients, and provides an opportunity to capture ECM activity in the subacute phase of repair.

Following the findings that measuring a combination of biomarkers may more appropriately capture ECM activity post-MI, we employed cluster analysis to identify whether patients could be partitioned based on ECM biomarker levels. Cluster analysis is an unsupervised classification technique that groups objects with similar characteristics together, and dissimilar objects separately^24^. This study carried out agglomerative hierarchical cluster analysis on the patient population. This is a common method utilised in cluster analysis, and involves the successive combination of cases into groups until an optimal grouping is identified^25^. In this study, AMI patients were clustered into three groups based on the levels of 10 ECM biomarkers. These findings suggest that the ECM response following AMI is not homogenous, and distinct ECM biomarker profiles can be identified within the patient population. These findings are of significance in this setting, as they suggest that patients with similar clinical characteristics and AMI presentations have altered ECM biomarker levels and these can be categorised using cluster analysis. More specifically, ECM biomarker levels are not uniformly increased or decreased within clustered groups, but instead display a combination of changes that could not be identified using a single biomarker approach.

For simplicity in understanding the potential biological relevance of clustered groups, we have described groups based on peak biomarker levels. Cluster Two had the largest number of peak biomarkers with increased levels of MMP-2, MMP-3, osteopontin, periostin and TIMP-1 when compared to other cluster groups. Cluster One had increased levels of MMP-8, MMP-9 and TGF-β1, while no peak biomarker levels were observed in Cluster Three. Levels of TIMP-4 and PINP were increased in Cluster One compared to Cluster Two, and but were not different when compared to Cluster Three. We suggest that ECM biomarker levels are representative of intra-cardiac changes in molecular function. As such, we postulate that patients in Cluster Two have greater ECM activity then patients in Cluster One or Three. We also suggest that patients in Cluster Three had decreased global ECM activity.

Deciphering what differences in ECM activity could mean for long-term adverse remodeling processes is not possible to ascertain in this study, as clinical indices of LV function were not routinely measured in this patient population. While we could not link ECM activity with direct measures of LV function, we examined whether clustering based on ECM profiles could differentiate patients based on two indices of cardiovascular risk. Peak TnT is a clinically useful biomarker and is routinely used for AMI diagnosis and estimation of infarct size^18^. In addition to its clinical utility, peak TnT is also associated with patient risk, with increased levels linked to LV remodeling and adverse long-term outcomes^26^. In this study, we have shown that peak hs-TnT is significantly elevated in patients from Cluster Two compared to Cluster Three, and is numerically higher compared to Cluster One. In addition, GRACE scores were significantly higher in Cluster Two when compared to other groups. Higher GRACE scores are associated with increased mortality risk at 6 months post-MI^27^, and this score has also been shown to hold value for predicting longer term mortality outcomes^19^. Combined, these findings suggest that clustered groups can differentiate patients into distinct risk categories based on ECM biomarker profiles despite similar clinical presentation. Identifying how ECM biomarker profiles relate to major adverse cardiovascular events (MACE) and adverse LV remodeling would be an appropriate next step.

Previous studies have demonstrated the ability of cluster analysis to capture disease heterogeneity^28,29^. Furthermore, the relationship between clustered groups and patient risk has been well-documented in the literature^29,30^. In a recent study of HIV-infected patients, Scherzer et al.^30^ demonstrated that patients could be partitioned into distinct groups based on the levels of serum-derived biomarkers only. Of the three groups, one was classified as a cardiac phenotype, one was classified as an inflammatory phenotype and one remained undefined. The two defined phenotypes were shown to be predictive of mortality in patients, demonstrating the significance of profiling patients based on biomarker levels. Similar to this study, we chose to create clusters based on biomarkers only. However, this is not the only approach to cluster analysis, as clinical variables and patient characteristics can also be included to strengthen patient subgroups^28,31^. Similar to the previous studies, we were interested in investigating the ability of biomarkers alone to group patients into distinct profiles and thus, clinical characteristics were excluded from cluster analysis generation.

This study includes some limitations that should be addressed. Firstly, the current study measured a number of circulating biomarkers that represent key processes in the ECM response post-MI. While these biomarkers are well-linked to repair processes and are known to change post-MI^5^, we cannot conclude for certain the measured levels directly reflect intra-cardiac composition. However, direct measures of biomarker levels within the heart are not possible without invasive procedures. Secondly, this study measured a number of correlations between biomarker levels without compensating for multiple comparisons. While this may influence the number of significant correlations captured in our analysis, this does not greatly influence the outcome of our results as we were interested in maximising the number of significant correlations observed. This study measured samples at a single standardised time point following AMI to reduce biomarker variation that might have occurred if we did not control for sampling time. While a single measure cannot capture temporal biomarker dynamics, this was not the purpose of the present study. Finally, we chose to use cluster analysis as a method for combining biomarker measurements. While this approach overcomes collinearity, selecting an appropriate cluster size can be difficult. To overcome this limitation, we employed the NBClust package^32^ in R that analyses optimal cluster size based on 30 well-established indices within the literature. This analysis provided an in-depth and extensive examination of optimal cluster size.

In this study, we have demonstrated that variation exists in the levels of ECM biomarkers measured in our patient population, and this can only be modestly described by patient characteristics and clinical presentation. We have shown complex interrelationships exist between ECM biomarkers using EFA, and measuring multiple biomarkers may more accurately capture the ECM biological process post-MI. Using cluster analysis, we identified three groups of patients which had distinct ECM profiles with differential patient risk. These clustered groups provide an opportunity to discriminate between patients during early myocardial repair. The clinical significance of these subgroups remains to be determined.

## Methodology

### Study population

Patients diagnosed with Acute Coronary Syndromes (ACS) and undergoing invasive therapy (coronary angiography ± percutaneous coronary intervention) at Wellington Regional Hospital between January 2012 and June 2018 were prospectively recruited into The Wellington ACS Registry. ACS was defined as having symptoms suggestive of myocardial ischemia for greater than 10 min, in conjunction with either troponin elevation or ≥ 1 mm of new ST-segment deviation or T wave inversion, as identified on an electrocardiogram in a minimum of two contiguous leads^33^. Patients were excluded from this registry if they had a platelet count less than 100 × 10^9^ /L, a known platelet function disorder, administration of a fibrinolytic agent within 24 h of enrolment or administration of a glycoprotein IIb/IIIa receptor agent within a week prior to enrolment. From this registry cohort, we included patients who had a primary diagnosis of acute myocardial infarction (AMI) and had blood samples collected three days post-hospital admission and prior to angiography. Patients were excluded from this study if they were documented as having had a previous AMI or a chronic heart failure (CHF) diagnosis, had an active malignancy, had pulmonary fibrosis, or had renal insufficiency determined by eGFR < 30 mL/min/1.73m^2^ or renal failure that was disclosed in the medical record. Patients with rheumatological diseases potentially influencing collagen turnover were also excluded from participation in the study (rheumatoid arthritis and osteoarthritis). Participation was voluntary and patients gave informed written consent at the time of recruitment. This study was approved by the Lower South Regional Ethics Committee (LRS/11/09/035) and the New Zealand Central Health and Disabilities Ethics Committee (16/CEN/68). All experiments were performed in accordance with the guidelines and regulations specified by these ethical committees.

### Data collection and blood sample

Demographic data, clinical characteristics, and aspects of clinical management were obtained prospectively from patient medical records. Whole blood was collected from AMI patients into sodium citrate tubes (0.109 M, BD Vacutainer, New Jersey, USA) three days post patient admission to hospital. Blood was collected from the peripheral vein using a 21-gauge needle or from the radial or femoral artery immediately after catheter insertion and prior to heparin administration in the cardiac catheterisation laboratory. Citrated whole blood was centrifuged at 1500× g for 15 min to generate platelet-poor plasma. Plasma was aliquoted and stored at − 80 °C for subsequent analysis.

### GRACE score

GRACE is a prospectively studied scoring system to evaluate the in-hospital and 6-month mortality in patients hospitalised with acute coronary syndromes (ACS)^27^. The GRACE score is calculated by assessing the following clinical parameters: age, heart rate, systolic blood pressure, creatinine, heart failure Killip class, cardiac arrest at admission, ST-segment deviation and abnormal troponin levels. All patients had a 6-month GRACE score calculated using Microsoft Excel software (Microsoft Corporation; Washington, USA).

### ECM biomarker measurement

A total of 10 ECM biomarkers were measured in this study. The rational for biomarker selection is shown in Table 5.

**Table 5:** Evidence for ECM biomarker role in ECM remodeling processes.

ECM biomarker	Rationale for measurement: evidence For role in ECM remodeling
MMP-2	Targeted gene deletion or pharmacological inhibition in mice can reduce left ventricular (LV) volumes^34^ and improve survival post-MI^34,35^
MMP-3	Associated with changes in LV function post-MI and indicated as a predictive marker of mortality and heart failure^36^
MMP-8	Indicated as a predictive marker of LV remodeling^11^
MMP-9	Murine targeted gene deletion protects against cardiac rupture^37^, reduces LV enlargement^8^ and reduces macrophage infiltration^37,38^ post-MI
Osteopontin	Gene deletion in mice leads to increased LV chamber dilation due to increased infarct expansion and decreased collagen accumulation post-MI^39^
Periostin	Mice with targeted gene deletion have increased mortality due to cardiac rupture post-MI^40^
PINP	Associated with parameters of LV function at 1 year post-MI as assessed by cardiac magnetic resonance^41^
TGF-β1	Pharmacological inhibition in mice increased mortality and LV dilation post-MI^10^
TIMP-1	Targeted gene deletion in mice leads to increased LV dilation and LV volumes post-MI^9,42^
TIMP-4	Targeted gene deletion in mice increased mortality due to LV wall rupture and reduced collagen synthesis^43^. Gene overexpression attenuated LV dilation, improved LV function and increased fibrillar collagen content post-MI in mice^7^

### ELISA quantification

Plasma concentrations of TIMP-1 and TIMP-4 were measured using commercially available sandwich ELISA kits (Human TIMP-1/TIMP-4 Duoset ELISA, R&D System, Minnesota, USA). PINP is a surrogate marker for collagen type I synthesis, and is cleaved during the post-translational modification of procollagen type I. PINP concentrations were measured using a commercially available ELISA kit (Human Procollagen I N-Terminal Propeptide ELISA Kit, Abexxa, Cambridge, UK). The level of TGF-β1 was measured in plasma samples using the Quantikine ELISA kit (Human TGF-beta 1 Quantikine ELISA Kit, R&D Systems, Minnesota, USA). All samples were measured in duplicate following manufacturer instructions, and absorbances were read using a Multiskan GO microplate spectrophotometer (Thermo Fisher Scientific, Massachusetts, USA). Intra-assay coefficient of variations ranged between 2.0% and 10.0%, and inter-assay coefficient of variations were between 1.9% and 11.3%. TIMP-4 and TGF-β1 concentrations were analysed by fitting a 4-parameter logistic curve to the standard analyte curves, while TIMP-1 and PINP concentrations were analysed by fitting a 5 parameter logistic curve.

### Human magnetic luminex quantification

The concentrations of MMP-2, MMP-3 and MMP-9 and the concentrations of MMP-8, osteopontin and periostin were measured in plasma samples using multiplex Luminex kits (Human Magnetic Luminex Kit, R&D Systems, Minnesota, USA), respectively. All samples were measured in duplicate following manufacturer instructions and measured on a Luminex 100/200 (Millipore Sigma, Massachusetts, USA). Intra-assay coefficient of variations were between 4.6% and 11.2%, and inter-assay coefficient of variations were between 7.3% and 13.2%. Experimental data was analysed by fitting a 5-parameter logistic curve to the standard analyte curves.

### Statistical analysis

Continuous variables were assessed for normality using the Shapiro–Wilk test. Parametric continuous variables were reported as mean ± standard deviation (SD) and non-parametric continuous variables were reported as median (interquartile range; IQR). Categorical variables were reported as frequencies (percentages). Univariate correlations were performed using Spearman’s Rank correlation. Statistical tests to compare medians of continuous and categorical variables were performed using Mann–Whitney U and Kruskal–Wallis H Test. Chi Square tests were used to compare categorical variables. Statistical significance was determined in this study by *p* < 0.05. All basic statistical analysis were conducting using either GraphPad Prism software version 7.04 for Windows (GraphPad Software Inc; California, USA) or SPSS v.24 (IBM; New York, USA). Visualisation of the correlation matrix was conducted in R version 4.0.2^44^ using the corrplot package^45^.

Exploratory Factor Analysis (EFA) was performed on 10 log-transformed ECM biomarkers using principle axis factoring with Direct Oblimin rotation in SPSS v.24. Eigenvalues were used to determine the number of factors extracted for this solution. Derived from matrix correlations between analysed variables, eigenvalues describe how well a single factor explains the variance in a solution. An initial analysis demonstrated that five out of 10 factors met Kaiser’s criterion of 1 (eigenvalue ≥ 1). A scree plot, which plots factors against respective eigenvalues, indicated a two-factor solution was most appropriate and this was further confirmed by parallel analysis^46^. In this study, we have presented all variables that have factor loadings > 0.3, which is an established factor loading cut-off in the literature^22^.

Cluster analysis was performed exclusively using ECM biomarker data and did not include clinical characteristics or patient risk factors. Prior to cluster analysis, biomarker data was log-transformed to normalise distribution and each biomarker was standardised to the same scale (mean = 0, SD = 1) to account for large variance between biomarkers which could influence cluster assignment. Subjects were partitioned using agglomerative hierarchical clustering using Ward’s method of minimum variance and the Euclidean distance metric. All statistical analysis associated with cluster analysis was conducted in R version 4.0.2^44^. Visualisation of the dendrogram was conducted using the factoextra package^47^, and identification of optimal cluster number was determined by the metrics in the NbClust package^32^.

## Supplementary Information

Supplementary Information.

## Data Availability

The datasets generated and analysed for this study are available from the corresponding author upon reasonable request.
