# Supplementary material for: A combined biomarker approach for characterising extracellular matrix profiles in acute myocardial infarction
Source: Sci Rep. 2021 Jun 16;11:12705. doi: 10.1038/s41598-021-92108-z (PMC8209070; doi:10.1038/s41598-021-92108-z)
Supplement: Supplementary file 1 — Supplementary Information. [file 41598_2021_92108_MOESM1_ESM.docx]

**Supplementary Materials**

**Full Title: A combined biomarker approach for characterising extracellular matrix profiles in acute myocardial infarction**

Author list: Morgane M. Brunton-O’Sullivan, BBMedSc (Hons)^1,2,*^, Ana S. Holley, PhD^1,2^ , Kathryn E. Hally, PhD^1,2,3^, Gisela A. Kristono^1,2^, Scott A. Harding, MBChB^2,4^, Peter D. Larsen, PhD^1,2,3^.

Affiliations: ^1^ Department of Surgery and Anaesthesia, The University of Otago, Wellington, 6242, New Zealand; ^2^ Wellington Cardiovascular Research Group, The University of Otago, Wellington, 6242, New Zealand; ^3^ School of Biological Sciences, Victoria University of Wellington, Wellington, 6141, New Zealand; ^4^ Department of Cardiology, Wellington Regional Hospital, Wellington, 6242, New Zealand. *morgane.brunton@postgrad.otago.ac.nz

**(r_s_=0.341, p<0.0001)**

**(r_s_=0.172, p<0.05)**

**(r_s_=0.206, p<0.05)**

**Supplementary Figure S1. Significant correlations between ECM biomarkers and peak hs-TnT.** All correlations were examined using Spearman Rank correlation and three biomarkers were significant (MMP-8, osteopontin and TIMP-1).

**(r_s_=0.250, p<0.01)**

**(r_s_=0.232, p<0.01)**

**(r_s_=0.304, p<0.001)**

**(r_s_=-0.208, p<0.05)**

**(r_s_=0.277, p=0.001)**

**Supplementary Figure S2. Significant correlations between ECM biomarkers and GRACE scores.** All correlations were examined using Spearman Rank correlation and five biomarkers demonstrated significant relationships (MMP-2, MMP-3, MMP-9, osteopontin, TIMP-4).

| **Supplementary Table S1. Relationship between ECM biomarkers and age, BMI and gender.** | | | | |
| --- | --- | --- | --- | --- |
| **ECM Biomarker** | **Age** | **BMI** | **Gender** | |
|  |  |  | **Male (n=107)** | **Female (n=33)** |
| MMP-2 | **0.295^++^** | -0.010 | 104.19 (93.92 - 134.21) | 117.09 (94.57 - 139.30) |
| MMP-3 | **0.216^+^** | -0.133 | **11.55 (8.44 - 17.04)** | **7.75 (5.52 - 9.68)***** |
| MMP-8 | -0.067 | -0.004 | 4.31 (1.69 - 7.46) | 4.0 (0.99 - 6.33) |
| MMP-9 | -0.140 | -0.003 | 17.47 (11.85 - 29.46) | 16.49 (11.65 - 25.91) |
| Osteopontin | **0.298^++^** | -0.028 | 34.95 (27.45 - 45.63) | 39.23 (26.10 - 49.51) |
| Periostin | 0.231* | 0.061 | 82.71 (67.34 - 101.78) | 91.40 (77.22 - 116.94) |
| PINP | **-0.197^+^** | -0.010 | **0.48 (0.26 - 0.87)** | **0.69 (0.37 - 0.69)*** |
| TGF-β1 | -0.018 | -0.010 | 4.45 (3.08 - 6.13) | 4.12 (3.06 - 6.20) |
| TIMP-1 | **0.194^+^** | 0.078 | **71.35 (53.64 - 82.2)** | **80.06 (63.14 - 94.77)*** |
| TIMP-4 | **0.429^++^** | -0.049 | **2.60 (2.02 - 3.33)** | **3.33 (2.10 - 4.67)*** |
| Continuous variables are reported as median (IQR). Spearman’s Rank correlation was used to measure the association between continuous variables. ^+^p<0.05, ^++^p<0.01. Mann-Whitney U testing was used to detect differences in ECM biomarker levels between male and female patients. ^*^p<0.05, ^***^p<0.001. All biomarker concentrations are recorded in ng/mL. | | | | |

| **Supplementary Table S2. The relationship between ECM biomarkers and hypertension** | | | |
| --- | --- | --- | --- |
| **ECM Biomarker** | **Hypertension** | |  |
|  | **None (n=78)** | **Diagnosed (n=62)** |  |
| MMP-2 | 103.63 (90.09 - 126.44) | 111.28 (96.95 - 146.11) | |
| MMP-3 | 9.82 (6.86 - 15.01) | 10.53 (8.11 - 15.18) | |
| MMP-8 | 0.39 (0.11 – 0.72) | 0.450 (0.18 – 0.78) | |
| MMP-9 | 16.98 (11.57 - 27.81) | 17.78 (12.30 - 28.94) | |
| Osteopontin | 34.85 (24.37 - 47.27) | 38.69 (29.90 - 47.48) | |
| Periostin | **80.31 (66.04 - 99.64)** | **94.08 (71.96 - 116.15)*** | |
| PINP | 0.55 (0.29 - 1.11) | 0.52 (0.33 – 0.91) | |
| TGF-β1 | 4.31 (3.04 - 6.18) | 4.55 (3.08 - 6.09) | |
| TIMP-1 | 71.35 (55.77 - 82.51) | 74.74 (52.26 - 91.25) | |
| TIMP-4 | **2.55 (1.94 - 3.28)** | **3.04 (2.19 - 3.76)**** | |
| Continuous variables are reported as median (IQR). Mann-Whitney U testing was performed to detect differences in ECM biomarker levels between patients with a hypertension diagnosis. *p<0.05, **p<0.01. All biomarker concentrations are recorded in ng/mL. | | | |

| **Supplementary Table S3. The relationship between ECM biomarkers and dyslipidaemia** | | | | |
| --- | --- | --- | --- | --- |
| **ECM Biomarker** | **Dyslipidaemia** | | |  |
|  | **None (n=64)** | **Diagnosed (n=76)** | |  |
| MMP-2 | 106.85 (94.34 - 128.14) | | 107.54 (94.03 - 140.52) | |
| MMP-3 | 9.95 (7.40 - 14.74) | | 10.92 (7.83 - 15.66) | |
| MMP-8 | 0.41 (0.17 - 0.70) | | 0.41 (0.11 - 0.77) | |
| MMP-9 | 17.27 (12.23 - 27.94) | | 17.32 (11.01 - 29.31) | |
| Osteopontin | 35.76 (23.90 - 46.63) | | 37.48 (28.68 - 48.75) | |
| Periostin | 82.52 (66.43 - 107.16) | | 86.29 (70.83 - 107.96) | |
| PINP | **0.38 (0.26 - 0.82)** | | **0.67 (0.42 - 1.15)*** | |
| TGF-β1 | 4.68 (3.42 - 6.18) | | 4.16 (3.03 - 5.88) | |
| TIMP-1 | 71.92 (54.75 - 84.79) | | 71.09 (53.07 - 86.49) | |
| TIMP-4 | 2.80 (2.02 - 3.69) | | 2.60 (2.05 - 3.40) | |
| Continuous variables are reported as median (IQR). Mann-Whitney U testing was performed to detect differences in ECM biomarker levels between patients with a dyslipidaemia diagnosis. *p<0.05. All biomarker concentrations are recorded in ng/mL. | | | | |

| **Supplementary Table S4. The relationship between ECM biomarkers and diabetes** | | | |
| --- | --- | --- | --- |
| **ECM Biomarker** | **Diabetes** | |  |
|  | **None (n=119)** | **Diagnosed (n=21)** |  |
| MMP-2 | 106.89 (94.99. - 134.21) | 111.12 (85.70 - 146.12) | |
| MMP-3 | 10.44 (7.75 - 15.16) | 9.37 (7.34 - 13.54) | |
| MMP-8 | 0.41 (0.13 – 0.73) | 0.52 (0.19 – 0.84) | |
| MMP-9 | 17.38 (12.03 - 29.08) | 14.73 (9.66 - 26.42) | |
| Osteopontin | 36.65 (25.74 - 45.63) | 39.59 (27.48 - 54.80) | |
| Periostin | 83.81 (67.49 - 104.49) | 95.91 (78.09 - 126.57) | |
| PINP | 0.54 (0.33 - 1.09) | 0.53 (0.17 – 0.87) | |
| TGF-β1 | 4.34 (3.04 - 5.83) | 4.75 (3.63 - 6.87) | |
| TIMP-1 | 71.80 (56.15 - 84.35) | 75.26 (52.93 - 102.90) | |
| TIMP-4 | **2.63 (1.99 - 3.39)** | **3.29 (2.31 - 4.16)*** | |
| Continuous variables are reported as median (IQR). Mann-Whitney U testing was performed to detect differences in ECM biomarker levels between patients with a diabetes diagnosis. *p<0.05. All biomarker concentrations are recorded in ng/mL. | | | |

| **Supplementary Table S5. The relationship between ECM biomarkers and AMI classification** | | | | |
| --- | --- | --- | --- | --- |
| **ECM Biomarker** | **AMI Classification** | | |  |
|  | **NSTEMI (n=103)** | **STEMI (n=37)** | |  |
| MMP-2 | 106.89 (94.16 - 131.80) | | 107.70 (91.07 - 144.15) | |
| MMP-3 | 10.18 (7.53 - 14.42) | | 9.92 (7.98 - 19.12) | |
| MMP-8 | 0.37 (0.11 – 0.73) | | 0.52 (0.24 – 0.79) | |
| MMP-9 | 17.25 (12.04 - 29.46) | | 18.03 (10.85 - 24.65) | |
| Osteopontin | **34.79 (25.74 - 43.97)** | | **39.99 (31.55 - 56.13)*** | |
| Periostin | 82.73 (67.49 - 104.49) | | 88.06 (70.82 - 114.49) | |
| PINP | 0.53 (0.30 – 0.88) | | 0.55 (0.31 - 1.15) | |
| TGF-β1 | 4.38 (3.03 - 5.83) | | 4.62 (3.61 - 6.20) | |
| TIMP-1 | 71.28 (52.64 - 83.29) | | 76.63 (58.58 - 91.91) | |
| TIMP-4 | 2.60 (1.99 - 3.37) | | 3.05 (2.25 - 3.93) | |
| Continuous variables are reported as median (IQR). Mann-Whitney U testing was used to detect differences in ECM biomarker levels between AMI classifications. *p<0.05. All biomarker concentrations are recorded in ng/mL. | | | | |

| **Supplementary Table S6. ECM biomarker levels for cluster assignment.** | | | |
| --- | --- | --- | --- |
| **ECM Biomarker** | **Cluster Assignment** | | |
|  | **Cluster One** | **Cluster Two** | **Cluster Three** |
| MMP-2 | 97.69 (86.5 - 126.4) | 139.4 (107.7 - 153.7)^†^ | 103.9 (91.5 - 154.3)^^^ |
| MMP-3 | 8.31 (7.08 - 11.57) | 15.19 (11.38 - 24.17)^†^ | 9.21 (5.52 - 12.25)^^^ |
| MMP-8 | 0.75 (0.44 - 1.02) | 0.38 (0.23 - 0.66)^#^ | 0.17 (0.08 - 0.71)^‡ †^ |
| MMP-9 | 27.89 (18.33 - 43.53) | 13.12 (10.79 - 18.21)^†^ | 14.29 (10.94 - 17.55)^†^ |
| Osteopontin | 33.78 (25.82 - 41.30) | 48.53 (38.90 - 62.32)^†^ | 31.23 (22.07 - 37.4)^^^ |
| Periostin | 82.52 (70.91 - 100.50) | 110.60 (92.26 - 132.60)^#^ | 71.17 (60.45 - 84.92)^^ *^ |
| PINP | 0.72 (0.45 - 1.38) | 0.36 (0.17 - 0.64)^†^ | 0.53 (0.30 - 1.30)^‡^ |
| TGF-β1 | 5.41 (4.26 - 6.79) | 4.13 (2.82 - 5.19)^#^ | 3.67 (2.74 - 5.20)^†^ |
| TIMP-1 | 71.75 (52.00 - 85.05) | 81.01 (65.48 - 94.35)^*^ | 61.97 (52.54 - 74.47)^§^ |
| TIMP-4 | 2.99 (2.14 - 3.75) | 2.97 (2.40 - 3.76) | 2.20 (1.53 - 2.59)^#^ ^^^ |

Kruskal-Wallis test with Dunn’s multiple comparisons was performed to identify significant differences between ECM biomarker levels in clustered groups. Symbols denote significant differences between clusters. *Cluster One (p<0.05), ^#^Cluster One (p<0.001), ^†^Cluster One (p<0.0001), ^‡^Cluster Two (p<0.05), ^§^Cluster Two (p<0.01), ^^^Cluster Two (p<0.0001). All biomarker concentrations are recorded in ng/mL.
